# SPARTAN: automated table detection and extraction from documents using advanced OpenCV heuristics and OCR techniques

**DOI:** 10.1038/s41598-026-44325-7

**Published:** 2026-03-25

**Authors:** Shlok Nandurbarkar, Archana Y. Chaudhari, Rahesha Mulla

**Affiliations:** Symbiosis Institute of Technology, Pune Campus, Symbiosis International (Deemed University), Pune, India

**Keywords:** Table detection, Table extraction, OCR, OpenCV, PDFs, Engineering, Mathematics and computing

## Abstract

The rapid growth of born-digital PDF documents has amplified the demand for fast, precise tabular data extraction on an industrial scale. State-of-the-art deep-learning approaches have high accuracy, but at the resource expense of substantial computational complexity, data-hungry training process and black-box incomprehensibility, confining deployment in the real world. In this paper, we introduce SPARTAN (Structured Parsing and Relevant Table Analysis), an entirely open-source, heuristic-based pipeline, with high-fidelity table detection and extraction and no model training or GPU requirements. SPARTAN mixes lightweight OpenCV image-processing modules: column whitespace analysis, boundary and text-based region segmentation and line segment cell parsing, with a modular OCR layer and optional post-processing hooks for LLM-driven schema mapping. We evaluated SPARTAN on more than 20 K pages of PCN-480 (Product Change Notification and Product Discontinuance Notification), scientific papers, certificates and datasheets and reported 0.94 precision, 0.91 recall and 0.93 F1-score, with 96.7% OCR character accuracy, processing a page in 2.8 s CPU time on an average, and requiring 1.2 GB peak RAM on the most demanding PDFs. Our model outperformed Tabula, Deepdoctection, TabbyPDF and EMbTTBF in accuracy and speed. It must be noted that this comparison was conducted against standard inference configurations for the learning-based baselines; a performance evaluation against highly-optimized, edge-device deployments is a consideration for future work. Its rule transparency effortlessly copes with borderless, nested and merged-cell layouts that easily outsmart classical heuristics, without incurring the resource cost of end-to-end neural pipelines. SPARTAN’s CLI-governed, swap-in-swap-out architecture encourages domain tuning, edge deployment and cloud-scalable REST service wrapping, making it a practical bridge between brittle rule systems and heavyweight AI for document-understanding pipelines. The work proves that well-crafted modernized heuristics, combined with high-quality OCR, can match or even outperform state-of-the-art deep learning while remaining within reach of small and medium enterprises, thus re-opening a critical gate to cost-efficient, explainable PDF table extraction.

## Introduction

In today’s era of digitization, the amount of data shared across the globe is unimaginable. It is estimated that around 182 zettabytes of data are shared daily over the internet. Since the internet infrastructure has improved across the globe, more and more people are joining the internet daily and thus in turn increasing the amount of data produced. This has also led to an increase in various types of data that are being shared from one system to another. This data type ranges from text, code, binaries, to multimedia like images, audio and video. Among this, one of the data types that has become one of the most shred type of data across is digital documents. Digital documents provide a way to wrap the data in human readable form at and share seamlessly around. The type of digital document that is more prominent is PDFs. The use of PDFs has become ubiquitous in companies, financial institutions, legal firms and educational organizations to store and share data. From scientific journals, legal bills, finance statements, educational topics, books are nowadays stored in PDF formats. PDFs are very human readable as they are designed for this exact purpose and remain uniform even if shared across various platforms. This robustness and uniformity make PDFs the most reliable medium to share complex data between two parties. Also, since the companies have started moving towards eco-friendly, carbon neutral models, this has given even more boost to the user of digital documents, especially PDFs to store data instead of paper wherever possible. That being said, the use of digital documents in industries and professional institutions is not new, but the sheer volume that is produced today has surpassed anyone’s imagination. Thus, this scenario has given rise to a new problem at the industry level which is extracting digital data automatically from these PDFs to be used in other workflows. Since the amount of the PDFs needed to be processed has grown to an extent that manual efforts to extract data have now become infeasible from a practical standpoint. The one of the strengths of the PDFs of being challenges in data extraction. Then general PDF to keep the file size small, doesn’t include any semantic details, this makes it difficult to identify the structure of the data in the PDF and extraction of relevant parts.

Although, in today’s date the extraction of plain text and identifying the relevant parts have become rather easy tasks, thanks to the advances in OCR technologies and NLP techniques. But when it comes to extracting tabular data from these PDFs, the challenges increase by ten folds. Tables in the PDFs still pose as one of the major challenges when it comes to automatic data extraction. As there is no defined structure of a PDF and the ambiguous nature of the table formats that vary vastly in their layouts, it just increases the challenge even more. Till now many different approaches have been proposed to address the challenges. Historically, it started with proposals of a few notable heuristic-based systems like TEXUS and TabbyPDF ^1,2^.

Traditional heuristic approaches showed their strength in being lightweight and approachable solutions. These approaches relied on visual cues like whitespaces, table borders, etc. to detect the table boundaries and thereby detecting tables. These approaches showed decent performance when it came to detecting and extracting simple tables, but they failed when the table complexities started to increase. Moreover, the heuristic-based approaches were very much prone to detecting false positives. These early roadblocks almost seized the research on heuristic-based approaches from then on.

Improved research in Machine learning and Deep learning technologies, gave rise to the use of learning-based approaches in table Detection. In 2019, TableNet was introduced, proposing an end-to-end model based on VGG-19 that jointly performs table detection and column segmentation using a multi-task learning approached^27^. TableNet achieved state-of-the-art performance on both the ICDAR 2013 dataset and Marmot datasets. This is considered as foundational work for learning-based approaches for the table detection and segmentation task which has further inspired research in this direction. For example, CascadeTabNet proposed a model that performs multi-stage object detection: in early stage it detects table regions and later it segments those regions^13^. This showed a joint approach to detect and segment using a single network instead of multiple. Later in 2023, GridFormer introduced Transformers based approach for grid-based representation for table structure recognition, where tables are interpreted through vertexes and edges on MxN grid^28^. TGRNet models tables as graphs where each represents a table cell, and edges represent relationships (such as adjacency or belonging to a row or column)^15^.

Although these models have shown exceptional performances in table detection and segmentation, most of them stop there and don’t go far enough toward data extraction. Apart from that these models are complex and computationally heavy and thus are not very easily accessible. Looking at these authors of^3^ introduced a hybrid approach called Tablext. It used a Convolutional Neural Network (CNN) to detect potential table regions in a document and used computer vision techniques to identify high level structures like rows and columns by incorporating line detection and morphological analysis, then a second CNN is used for detecting bounding boxes of the individual cells. This showcases that the challenge of table detection has been a major research domain which still seeks further iterations.

### Background

Historically, many various approaches have been applied to meet the challenge of table detection and extraction from PDFs. All the approaches have had their pros and cons. Heuristic approaches were applauded for their lightweight nature and transparency, whereas on the other hand, deep learning-based models have shown exceptional performance and accuracies at the cost of computational resources. Hybrid approaches have tried to find a fine balance between both approaches. But given all this, none of these approaches have found their way as practical solutions in industrial or other settings. Deep learning approaches, despite being competent, have struggled to mark themselves practical approaches due to their complex nature. This might be because a complex solution for seemingly rudimentary task of table detection and data extraction does not sound feasible to a general user. It might feel like using a sword for cutting vegetables, yes it does the job, but is it an ideal solution? Recently, LLM and Transformer based models like ChatGPT from OpenAI have lent their services available to the user as SaaS on cloud infrastructure. But they too come with their fair share of biases, ambiguities, connected costs and security threats. Moreover, this kind of service is mostly limited to large organizations who can afford their subscription costs. Small to medium sized institutions still struggle to latch on a reliable solution. The current data suggests that a lot of research transitioned towards deep learning approaches very quickly, riding on the AI-ML boom, leaving the heuristic-based landscape somewhat unexplored. Thus, we propose a new heuristic-based approach called SPARTAN (Structured Parsing and Relevant Table Analysis) to address these issues. SPARTAN aims to be a lightweight, practical and industry focused solution for detection of table and d`ata extraction. These showcases that carefully crafted heuristics and modular approach could prove to be feasible for widespread adoption. Although SPARTAN is not guaranteed to work on scanned PDFs or scanned handwritten documents as of now, it shines on the general-purpose digital-born PDFs.

### Motivation

SPARTAN was developed because of increased demand for an automated solution for table detection and extraction from PDF in industries. It also tries to be an easy to access and use solution for an average user for most of the standard PDF formats. SPARTAN’s lightweight design was designed to counter resource intensive solutions. By developing a lightweight, CPU-friendly and fully modular system, SPARTAN looks forward to broader adoption, particularly for individuals and small organizations. Its design highlights the robustness against diverse document types, easy customization of pipeline stages and full explainability for practical auditing, bug fix and domain adoption. SPARTAN broadens its spectrum by not limiting itself only to table detection but approaching document understanding holistically, supporting integration of LLMs, knowledge graphs by making sophisticated document automation possible. We aim to make SPARTAN open source and available as a python library.

## Related work

Automatic table detection and extraction from paper documents has been widely researched in the literature and has produced an enormous diversity of methods. Existing methods broadly fall into heuristic-based methods, hybrid methods, and deep learning models. Within each category, different methodologies exist along with varying benefits and drawbacks as observed in the plethora of prior work in literature. We survey notable systems in each of these categories in this section – from heuristic-based systems such as TEXUS and TabbyPDF, to hybrid systems such as TablexT, to end-to-end learning-based systems – and consider the performance trade-offs.

### Heuristic based approaches

Traditional and early methods for table extraction are based on rule-based heuristics and layout analysis. These methods employ document layout characteristics such as lines, whitespace, text alignment, and font style to identify table areas and distinguish between their internal organization. TabbyPDF^2^, for instance, is a heuristic-based system for table detection within PDF documents and structure recognition, employing textual and geometric layout characteristics to identify tables and deduce cell organization with no machine learning components. Likewise, the TEXUS system^1^ provides an entirely automatic pipeline for table processing within PDF documents, employing specialized table segmentation and interpretation algorithms that are layout independent. Heuristic-based methods like these are not usually trained on data and are computationally efficient, so are quick and easy to run. However, their performance can depend on strong assumptions concerning table format (e.g., ruling lines or consistent column alignment). Thus, rule-based extractors can be brittle; they struggle with complicated or irregular layouts, are weak when tables are displayed as images or do not have clear boundaries, and usually require extensive tuning for every new document class.

### Deep-learning based approaches

In the last few years, the trend in table recognition and detection has shifted towards data-dependent, deep learning-based approaches. These approaches treat table identification as object detection or image segmentation and use convolutional neural networks and related models to detect tables directly from document images. For example, TableNet^27^ proposes an end-to-end CNN model that performs both table region detection and table structure recognition (breaking up a detected table into its individual rows and columns) within one model. Similarly, CascadeTabNet^13^ employs a cascade of deep object detectors to enhance table detection and then predicts the table structure at the cell level using advanced segmentation networks, which achieves state-of-the-art accuracy on benchmark datasets. Deep learning models have demonstrated outstanding performance, especially on complex documents and cases where tables are visually embedded (scanned documents or images of tables) that confound simpler heuristics. They can learn strong features to handle a wide variety of table styles, even when ruling lines are missing or layouts are extremely irregular, provided adequate training data is present. The biggest disadvantage, though, is that these models require large, annotated datasets and considerable computational power. Training such networks takes a long time and necessitates GPU hardware, and even inference may be relatively slow compared to rule-based methods. In addition, purely deep learning-based solutions are “black boxes,” and it is difficult to interpret failures; they can generalize poorly to documents that are dissimilar to the training distribution or exhibit bias inherited from training data. In short, although deep learning solutions push the accuracy of table extraction to unprecedented heights, they do so at the cost of data dependency and computational overhead. Therefore, some researchers have examined combined or cut-down methodologies (as mentioned earlier) to find a balance between efficiency and accuracy.

### Hybrid approaches

To mitigate the brittleness of pure heuristics without paying the full cost of deep learning, several works have combined the two. TablexT^3^ is one such hybrid neural-network and heuristic-based table extractor. It employs a multi-stage pipeline: a custom convolutional neural network (with a YOLO object detector inserted) first finds and extracts candidate table regions in the document, then standard image processing algorithms are employed to determine the table’s high-level structure (e.g. splitting rows and columns), and finally a second CNN model detects the exact cell boundaries before applying OCR to the cells to extract cell content^3^. By delegating simpler layout tasks to fast image processing code and reserving deep neural networks for the harder recognition tasks, TablexT shows how hybrid systems can take advantage of the strengths of both. Other researchers have used similar strategies, using heuristic page layout analysis in pre- or post-processing to assist neural models – e.g. by restricting table searches to likely areas or eliminating false positives – which has been shown to improve accuracy and reduce errors in table extraction systems. Such hybrid systems tend to perform better than purely rule-based systems on challenging inputs (e.g. tables with no obvious lines or noisy backgrounds) and can generalize better across a range of formats without being trained on as much data as fully end-to-end systems. But the inclusion of learned components means hybrid systems are still dependent on annotated data and model training to some extent, and their pipelines become more complex and modular, so careful engineering is required to ensure all the components work together in harmony.

### Research gaps and observations

While many table detection and extraction algorithms have been presented, there are still a few significant research gaps in current work. First, the area increasingly relies on deep learning models, which also possess a set of practical limitations. Most current state-of-the-art methods are greatly dependent on powerful neural networks^4,5^ this requires enormous, annotated training data and large computational capacity for training and inference. The need for enormous amounts of annotated data is a significant hurdle, as high-quality table annotations require time to create, and some areas (e.g. historical text or specialized industries) have little training data. The high computational expense of deep models also renders such solutions effectively infeasible in resource-constrained environments; real-time or on-device table extraction is effectively infeasible with current deep learning pipelines due to processing and memory requirements. Additionally, deep models can absorb biases from training data, causing varying performance or failure on tables with styles or contents less represented in the training data. As an example, a model predominantly trained on English financial tables will fail on scientific tables or on documents in another language with another formatting regime. These biases and generalization flaws indicate the risk of exclusive dependency on data-driven models.

Conversely, heuristics-based solutions have attracted relatively less research attention in the past few years. With increasing interest in machine learning, there has been decreasing research in enhancing rule-based table extraction systematically. This is a critical shortcoming: although heuristics-based solutions may have some limitations, they have advantages in scenarios where deep learning is not optimal—e.g., scenarios with limited data availability, rapid processing needs, or the requirement for explainable decision-making. Literature lacks examination of how much lightweight solutions can be enhanced with modern methods of image analysis. There is potential to strengthen heuristics (e.g., enhancement of line detection, shape assessment, and textual contextual reasoning) to make them more reliable; however, few recent studies address this, leaving part of the potential solutions underdeveloped. In general, there is not a widely known lightweight, modular solution that can compete with the flexibility of deep learning models without abandoning their high demands. Most of the available heuristic tools are proprietary, narrowly specialized in application, or unoptimized for wide use, thus contributing to the unavailability of table extraction technologies for many end-users. Researchers or practitioners without data collection and computer infrastructure access required for deep learning are left with the use of outdated or brittle rule-based systems.

The identified gaps are necessitating methodologies that strike a balance between practical efficacy and broad applicability. The above challenges are addressed by this thesis in the form of the SPARTAN. It is particularly geared to solve the identified shortcomings of being lightweight and not depending on large training data or GPU processing, hence being deployable in real-world environments and on open-source hardware. It is important to note that while SPARTAN showed superior performance, the comparison with learning-based baselines was made against their standard configurations, and an evaluation against highly optimized, edge-device-specific deployments for low-latency comparison is a direction for future work. Its modularity splits the process into replaceable components (e.g., preprocessing, table region detection, structure parsing, and text recognition), enabling fine-tuning and adaptability to diverse document types without retraining a single, monolithic model. This modularity also lends itself to easier transparency and maintainability, with each step’s logic being understandable and optimizable independently. Furthermore, by leveraging heuristics derived from knowledge of document layout, SPARTAN avoids the bias problems inherent in learned models and delivers consistent performance across different document styles (barring the correctness of the underlying assumptions, which are explicitly chosen to be general). Above all, SPARTAN is an open-source system, directly tackling the problem of access—it can be freely used and extended by the community, thus lowering the barrier to effective table extraction technology. In brief, the research gaps in the existing literature on table extraction—ranging from overdependence on data-greedy deep learning to the ignoring of effective heuristic solutions—are exactly the gaps SPARTAN is designed to fill. Building on the strengths of heuristic image analysis and the limitations of previous approaches, SPARTAN offers a practical and new solution to achieving accurate table detection and extraction with fewer dependencies, thus bridging a major gap in the field of document analysis techniques.

## Methodology

The process of SPARTAN has been designed with precision to cater various types of table layouts by our unique “pre-text, “inter-text” and “post-text” region segmentation approach and identifying various components within the table itself like the table header, column headers and nested column headers, spanning cells along with various corrective steps like OCR auto-correction step which identifies the potential mistakes made by the OCR model based on the trend in text for that given column and correcting the values accordingly. Our methodology has been designed while keeping a modular architecture in mind. For example, changing the underlying OCR model with ease and changing the scope of the OCR model from single cell OCR to multi-cell at a time. We also have designed a simple CLI interface, which also allows users to include or exclude any step of the pipeline in the extraction process. This gives user control over the overall processing of their concerned document, while keeping the workings transparent and easily customizable. Since SPARTAN is not a learning-based model, it has been designed while taking the references of various layouts at best, thereby avoiding any requirement of training process, large training datasets and biasness towards a particular type of data. Since majority of the pipeline is heuristic based comprising various intricate image analysis steps, computational efficiency has become one of the major strengths of SPARTAN. This section goes through the testing datasets that were used, a high-level description of overall process and description of each step involved in detail.

### Testing dataset summary

**Fig. 1:**
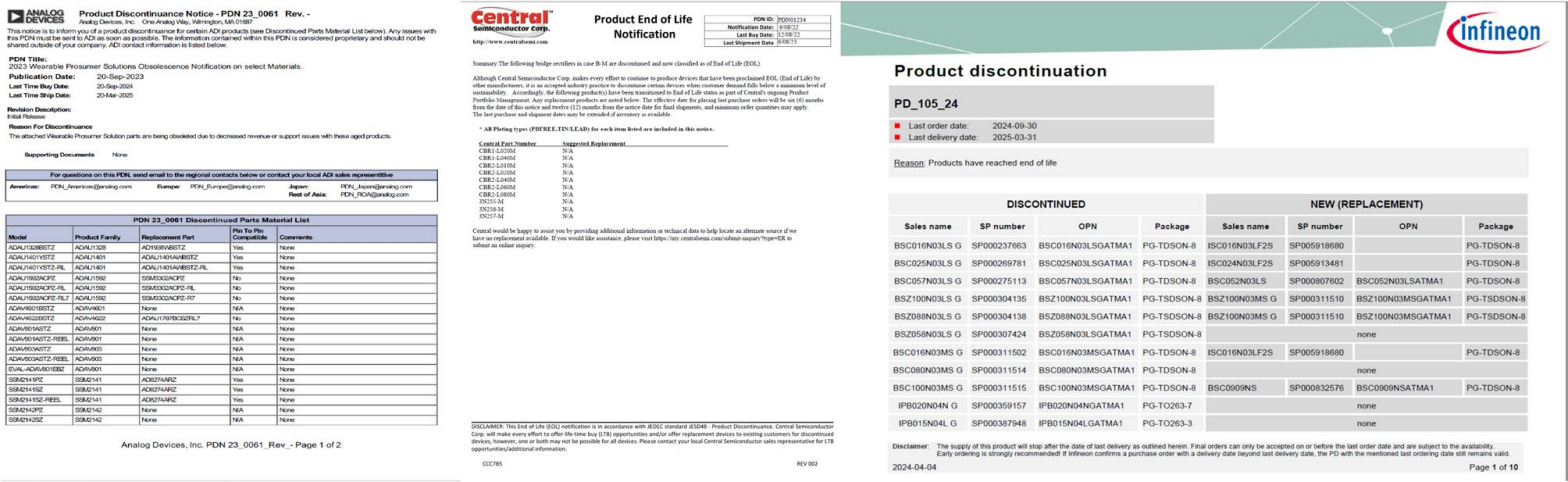
Examples of the PCN-480 dataset showcasing unusual table layouts.

The test was designed to thoroughly challenge SPARTAN with the diverse types of documents it will most commonly see in production use as depict in Table 1. PCN-480 dataset offers most pages and most structure variations, i.e., border-less pages, sporadic nested headers and vendor specific behavior, representing the electronics change-notice workflow that motivated the development of this work as shown in Fig. 1. To avoid overfitting to a single domain, a smaller selection of scientific papers introduces a high frequency of border-less formats typical of academic PDFs, and the subset of certificates and datasheets is predominantly made up of scans and stamped documents.

**Table 1:** Testing dataset metrics and summary.

Corpus	Pages		Border-less tables	Nested header	Source
PCN-480 (Product Change Notification and Discontinuance Notices)^29^	18,642	1927	23%	7%	Texas Instruments, Analog Devices, Vishay, Onsemi, Intel, Infineon, Central Semiconductors, Mini-Circuit, 3 M and more
Scientific-Journals (random 2023 sample)	1200	214	48%	2%	arXiv & IEEE PDFs
Certificates and Datasheets	390	97	31%	0%	UL, CE, ISO downloads

These PDFs presented a variety of table types, posing a significant challenge for the SPARTAN model. It must also be noted that SPARTAN was primarily refined through internal testing on partial PCN-480 dataset. Therefore, one might argue that SPARTAN could have an advantage over other models when tested on the PCN-480 corpus. However, it has been ensured that the PDFs used during the refinement and development of SPARTAN were not reused in testing, although similar layouts might have appeared in the testing corpus. That is why including additional scientific journals, certificates, and datasheets was an important aspect of the testing dataset. Furthermore, manufacturer sources such as ONSEMI and 3M were never utilized during the refinement or development of SPARTAN; thus, they represent entirely new data types for SPARTAN testing. All these considerations were carefully implemented to ensure an unbiased testing environment.

### Process overview

**Fig. 2:**
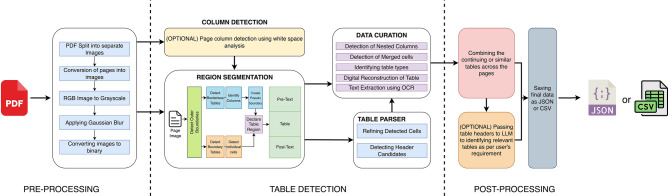
Overview of process flow.

The Fig. 2 provides a high-level overview of the SPARTAN pipeline and how the data is passed from one module to another. The following diagram gives an overview of the whole process at a glance. All the steps in the process have been discussed in detail in the upcoming sections.

As seen from the Fig. 2, the SPARTAN pipeline starts with a Pre-Processing step, followed by an option column detection step, then it moves to the region segmentation where the core logic of SPARTAN resides. After processing the data in the region segmentation step the data is passed to both table parsing and data curation modules. The data curation module waits for the table parsing module to complete its processing and pass its data to the data curation module itself. Once the data curation module receives data from both the sources, it does the job of detecting other features from the table and prepares a digital copy of the table for OCR. Then once the OCR is done the extracted data along with the page information is passed to the Post Processing function, where similar tables or continuing tables are merged across the different pages. Optionally one can incorporate LLMs in the post processing step for building knowledge graphs or extracting specific details out of tables or the text of the surrounding regions of the tables. The following table mentions the technical specifics of each module in a structured manner.

SPARTAN has been developed using Python programming language. Al the modules of SPARTAN are being controlled by the parent module “spartan.py”. The CLI Flags mentioned in the above table can be passed as arguments to the “spartan.py” to control its behavior, essentially the flags passed means that particular module in the pipeline should be included, if not passed then that module should be bypassed during the processing. Although, it should be noted that wrong configuration of inclusion and exclusion of some modules can create logical errors during the processing of the PDF. For example, if the table parsing step is skipped before the data curation step, then the data curation step may throw an error or would not behave as expected. Making the whole pipeline yield potentially useless output. Thus, some steps have been made implicit in the pipeline, and they are mandatory to be included. Finally, in a nutshell, the SPARTAN modules take input of a PDF file and output a JSON file containing table data in structured manner and all the other surrounding text. The Table 2 summarizes the key operations of each module along with their CLI-flags and I/0.

**Table 2:** Technical overview of complete process.

Stage	CLI Flag	I/O	Key operations
Pre-Processing	--preprocess	PDF → RGB images	300 dpi rendering (pypdfium2) optional Canny overlay
Column Detection	--column	Page image → column- segments	Vertical run-length analysis and whitespace RLE
Region Segmentation	--region	Column/Page image → segmented images	Boundary-based + text-based table localization; pre/inter/ post-text tagging
Table Parsing	*Implicit*	Segmented image → cell boxes	LSD line detector, cell-merge logic, nested column discovery
Data curation & OCR	--ocr-engine & --chunk-size	Cell boxes → raw JSON	Single cell vs. chunked OCR, error-correct, header promotion
Post-Processing	*Implicit*	Page JSON → document JSON	Table merge across pages, LLM schema mapping

There is also a “--save" CLI Flag, that lets user save the consequent output of each stage in the local memory, if the user want transparency for the process being carried out at each stage, for auditing the process or for debugging.

### Pre-processing

**Fig. 3:**
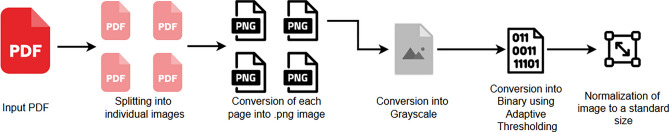
Pre-processing pipeline.

Pre-Processing is the foundational step that begins the pipeline by taking in the input PDF and splitting each page of PDF into separate pages and then converting them into image (.png files). Once the images are acquired, the first operation performed is grayscale conversion. This step removes redundant color information and reduces computational load without sacrificing critical spatial features as shown in Fig. 3.

After converting to grayscale, we apply adaptive thresholding to convert the image into a binary representation. Adaptive method is preferred rather than global thresholding because adaptive method can handle varying illumination levels and document shading effects. After conversion into binary, the difference between the tables, texts and the background increases thereby aiding the subsequent segmentation steps.Finally, we apply normalization over each page image to ensure that all the pages maintain a standard size format. This normalization is important as the PDF can originate from varying sources and have different dimensions. The result of this complete pre-processing step is a stable image that could be a reliable foundation for the subsequent complex steps.

### Table detection

The Table Detection step is obviously the most important part of our pipeline. The complete table detection has many complex sub-routines that ensure that each aspect of the table detection process is addressed thoroughly yielding a high-quality table detection result. These steps include the optional column Detection, Region Segmentation, Table Parsing and Data Curation.

#### Column detection

Column detection is an important step if the input PDF has a columned layout, like the one that is generally seen in scientific literature. If not handled carefully then all the upcoming steps could render useless results.

**Fig. 4:**
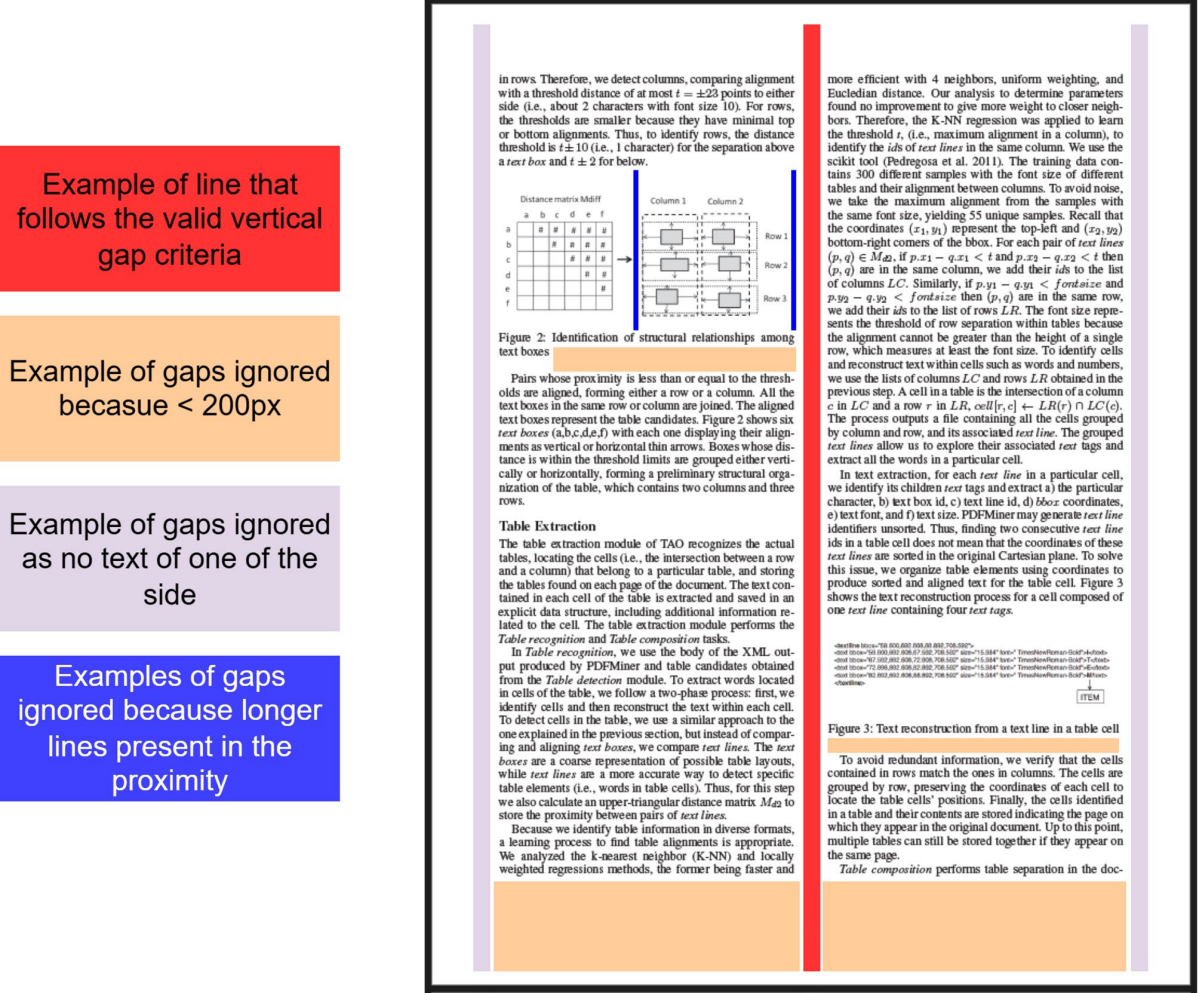
Column detection step.

This step works by analyzing all the continuous vertical white space i.e. analyzing all the different vertical gaps. Then all the gaps whose vertical length is less than 200px are ignored. This helps in eliminating false positives. Then all the other vertical gaps are eliminated that don’t follow our criteria which is “The vertical gaps must have text at a distance of at most 15px from the either side of the gap”. In simple words there should be text on both sides of the vertical gap for it to count as valid vertical gap. This helps in eliminating the boundary margins to be falsely counted as valid vertical gaps. If a longer vertical gap is available in the proximity of the given vertical gap, then the smaller one is ignored. Then all the vertical gaps that share the same x-co-ordinates are merged if they are less than 15px closer to each other. Finally, the page image is split into different column segments based on these vertical gaps that were identified as shown in Fig. 4. Then all these column segments are treated as individual page images in the subsequent steps.

Thus, column detection serves as a simple yet powerful step in the pipeline to correctly identify the columned layout of the document and process it accordingly. But this step is kept as an optional because this step is relatively time-consuming and it is not required if the user already knows that their concerned PDF is not a columned layout. This step tends to identify the false positives in case of single layout documents having borderless tables.

#### Region segmentation

This step is the crux of the whole pipeline as if this step fails to misidentify the segments correctly then all the subsequent steps would yield wrong results. This step takes in the page image or the column segment image from the column detection step (if opted) as an input. The purpose of this step is to identify the potential table region and then splitting the page into “Pre-Text”, “Post-Text”, “Inter-Text” and “Table” itself. The definition of each is as follows: Pre-Text: The region before the first table of the page. Post-Text: The region after the last table of the page. Inter-Text: The region between the two tables, if there exist multiple tables on a single page. Table: The identified table regions itself.

**Fig. 5:**
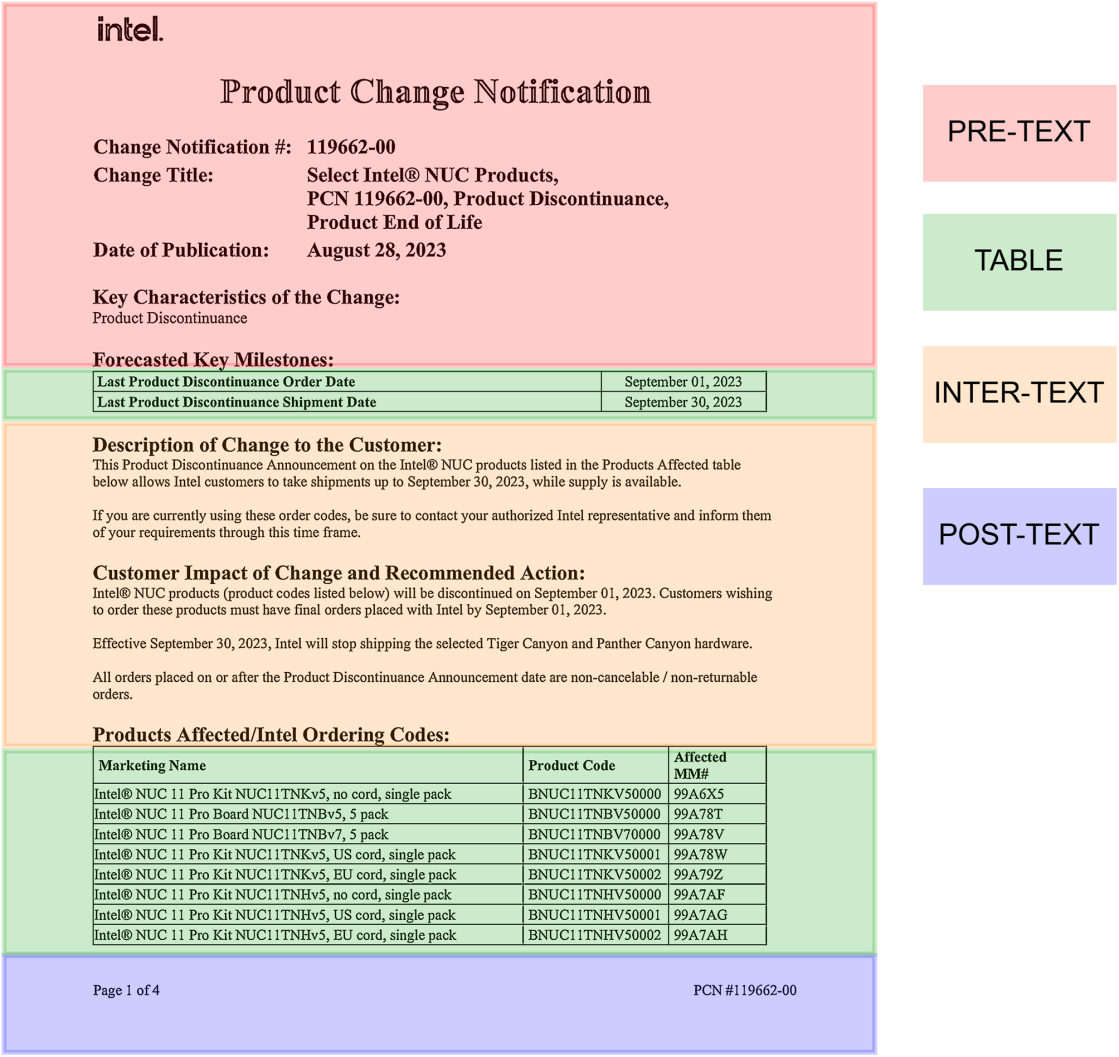
Example of region segmentation output.

As depict in Fig. 5 the unique approach allows us to isolate the table regions during the further processing simplifying the steps that involve identifying complex features of the table and yielding high-quality data extraction. The rest of the segments like “Pre-Text” helps in getting information about the context of the tables in that page or some other meta data regarding the tables or the entire document and helps in finding out if the next table is being continued from the last page. The “Inter-Text” helps find out if there is any linkage between the two subsequent tables or if the context of the next table is available. The “Post-Text” segments help to find if there is any footer information regarding the table or the given page or given document and might help to determine if the previous table is being continued to the next page.

Before reaching the successful table region detection, many intricate steps are taken care of which ensure that the table region detection is more robust and high-quality table detection is passed to the next step. These intricate sub-steps include cropping of outside boundaries, detection of tables based on boundary, detection of tables based on text arrangement (for border-less tables), unification of tables and finally segmentation.

##### Cropping outer boundaries

**Fig. 6:**
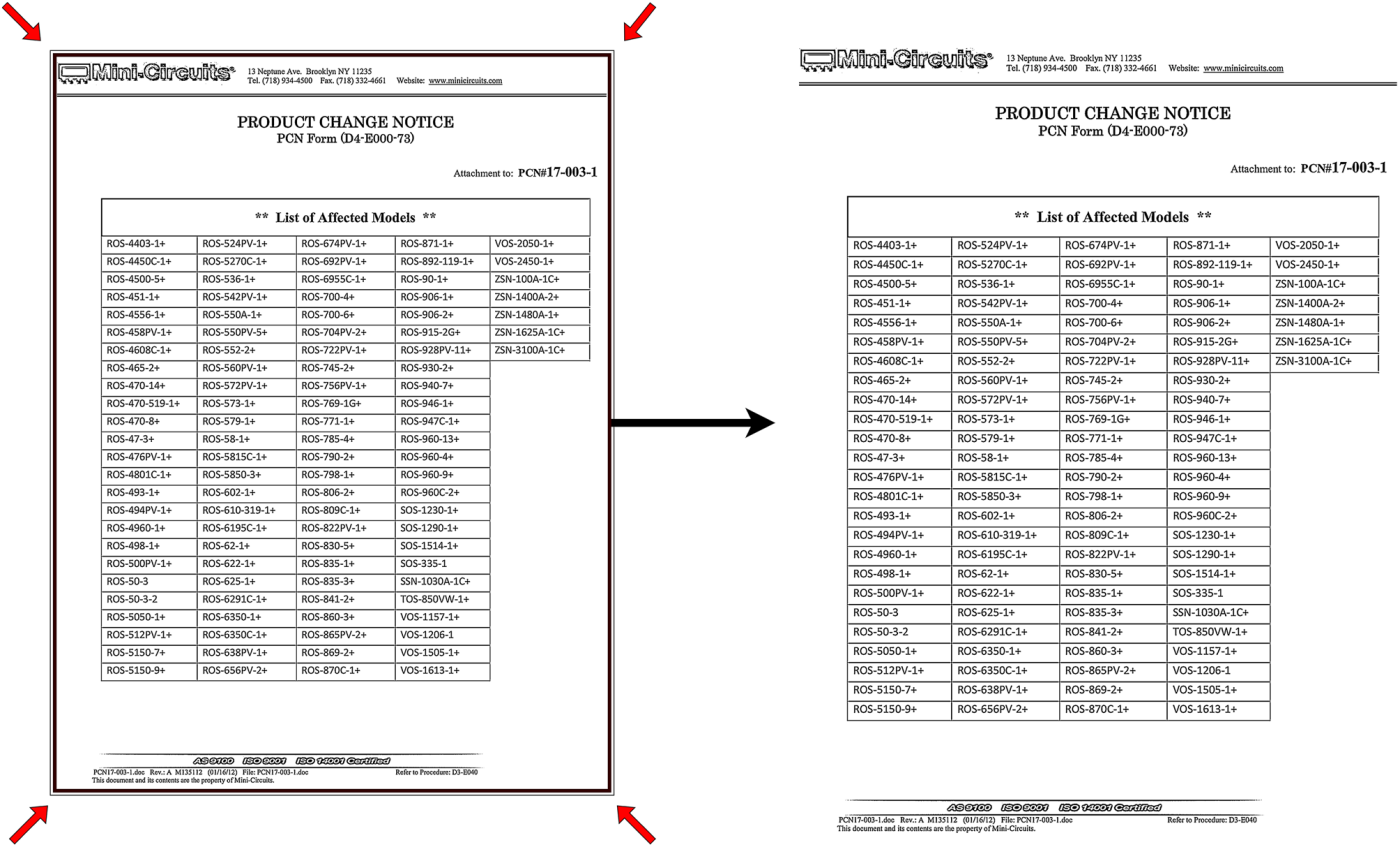
Cropping outer boundaries.

This step looks for the largest four-sided contour on the page, if that contour covers more than or equal to 75% of the page, the function chops it off with a 25px safety margin on every side as shown in Fig. 6. Many PDFs are rasterized with a black border or scanning frame. Therefore, this step is important so that the outer boundary does not interfere with the table detection logic resulting in whole page being identified as a table.

##### Boundary based detector (for tables having visible boundaries)

**Fig. 7:**
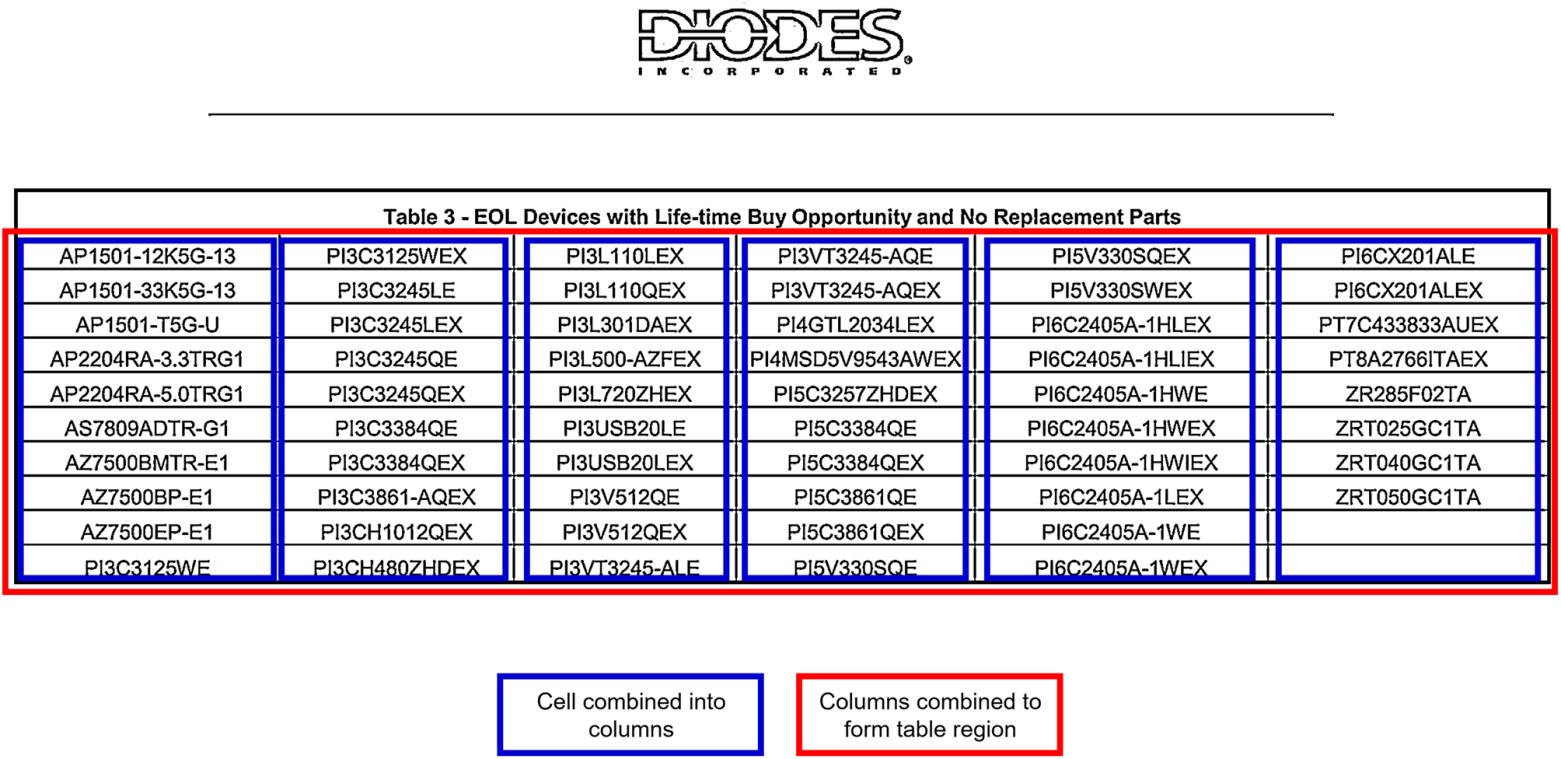
Detection of boundary tables.

This step looks for tables that have visible boundaries by utilizing line extraction, dual path → dilation and no-dilation, contour detection for cell finding, sorting detected cells based on the co-ordinates, forming columns based on cell arrangements and finally combining columns to define table regions as shown in Fig. 7.

The Table 3 summarizes the technical details of the boundary-based detection step mentioning the key operations happening in this step and revealing more insights about table region selection criteria.

**Table 3:** Technical overview of boundary based detector.

Stage	Key operations	Tunables
Line extraction	Morphological open with kernels 25 × 1 (horizontal) & 1 × 25 (vertical)	–
Dual paths	Run the same logic with and without an extra 3 × 3 dilation; keep whichever path yields more cells	use_dilation flag
Cell finding	Contour → approxPolyDP → keep 4‑sided, convex boxes; size filter w > 100 & h > 20	–
Table clustering	Sort cells top‑to‑bottom/left‑to‑right → cluster rows whose top edges are within 20 px (cell_gap_threshold) → compute union bounding box	cell_gap_threshold = 20
Table acceptance	Final bounding box must satisfy w ≥ 500 & h ≥ 100	–

##### Text based detector (for table not having visible boundaries)

Once the boundary-based detection process is complete, the text-based detection step is activated. The main purpose of this step is to look for tables that have no visible boundaries and are formed purely by textual arrangements. Here we look for words that are vertically stacked over each other. For checking vertical stacking, we first check if the left edge of the words is aligning over each other by checking the x co-ordinates of the left edge of each word with tolerance of 5px. If no group of words are found to be following this trend, then we check for the center alignment of the text. For all the words that are following this trend are considered to be part of a single column.

Then we check if there are any such columns that are adjacent to each other on y-axis, i.e. we check if the top of edge of two columns shares the same y-axis co-ordinates with tolerance of 5px. If there are at least two such columns following these criteria, then we declare that entire region as a valid table region.

**Fig. 8:**
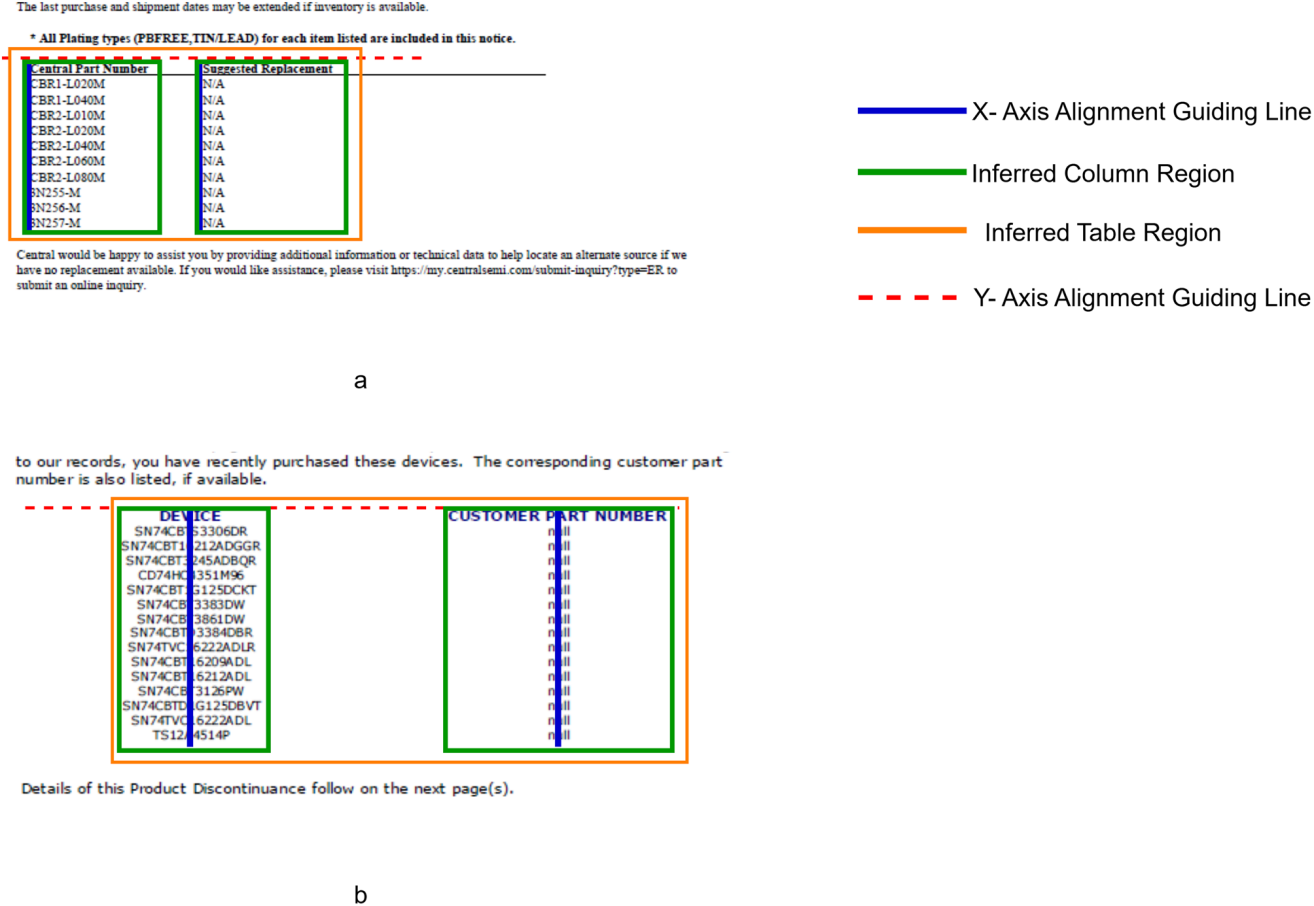
Depiction of table region recognition for boundary less tables with two strategies: (**a**) Left edge alignment. (**b**) Text center alignment.

After the successful detection of the table region, by utilizing the column co-ordinates and the table region coordinates, we create pseudo boundaries around the text and then this new image with boundaries is passed to the next modules in the pipeline, so that this boundary less table is treated as a regular boundary table in the subsequent tasks and we don’t need to create separate logic to handle the boundary less table in any of the subsequent tasks as shown in Fig. 8.

##### Table unification

The boundary-based table is also prone to pass the boundary less table detection logic, thus if the detected boundary-less table is overlapping the boundary table, then we discard the boundary-less table detection data, as obviously both of the logic are pointing towards the same table. If there is no overlapping whatsoever, then we keep both tables.

##### Region segmentation

**Fig. 9:**
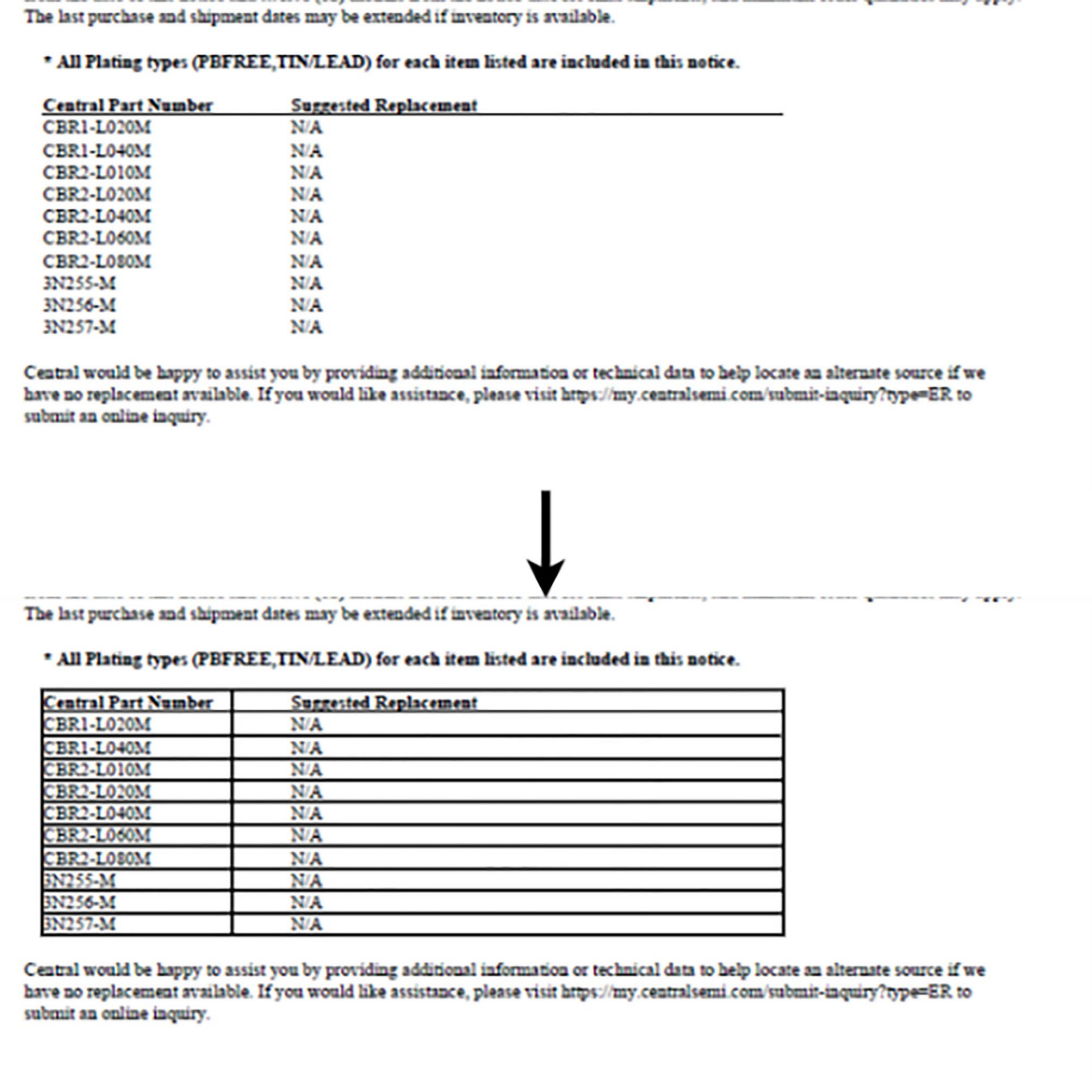
Conversion of boundary less table to boundary table using the column region and table region data.

Finally, based on the detected table region, we segment the page image into Pre-Text, Post-Text, Inter-Text (Inter-Text occurs only if there are multiple tables detected on the same page) and the Table itself as discussed in the beginning of this Region Segmentation section as shown in Fig. 9.

#### Table parsing

Even though the region segmentation step does quite a heavy lifting when it comes to detecting cells, columns and table regions, but still sometimes a few false positives slip through those filters. Also, the cell data derived from that step is prone to some errors due to breakage in boundaries of the tables due to poor quality of the original document or due to any ghost lines that led to formation of wrong cells at the first place. Region segmentation captures the overall region hinted by these components therefore these minor errors don’t cause much of a problem while deciding the table region in the region segmentation step, but when it comes to extracting complex features out of the tales and extracting data from these tables, it certainly requires some extra refinement.

**Fig. 10:**
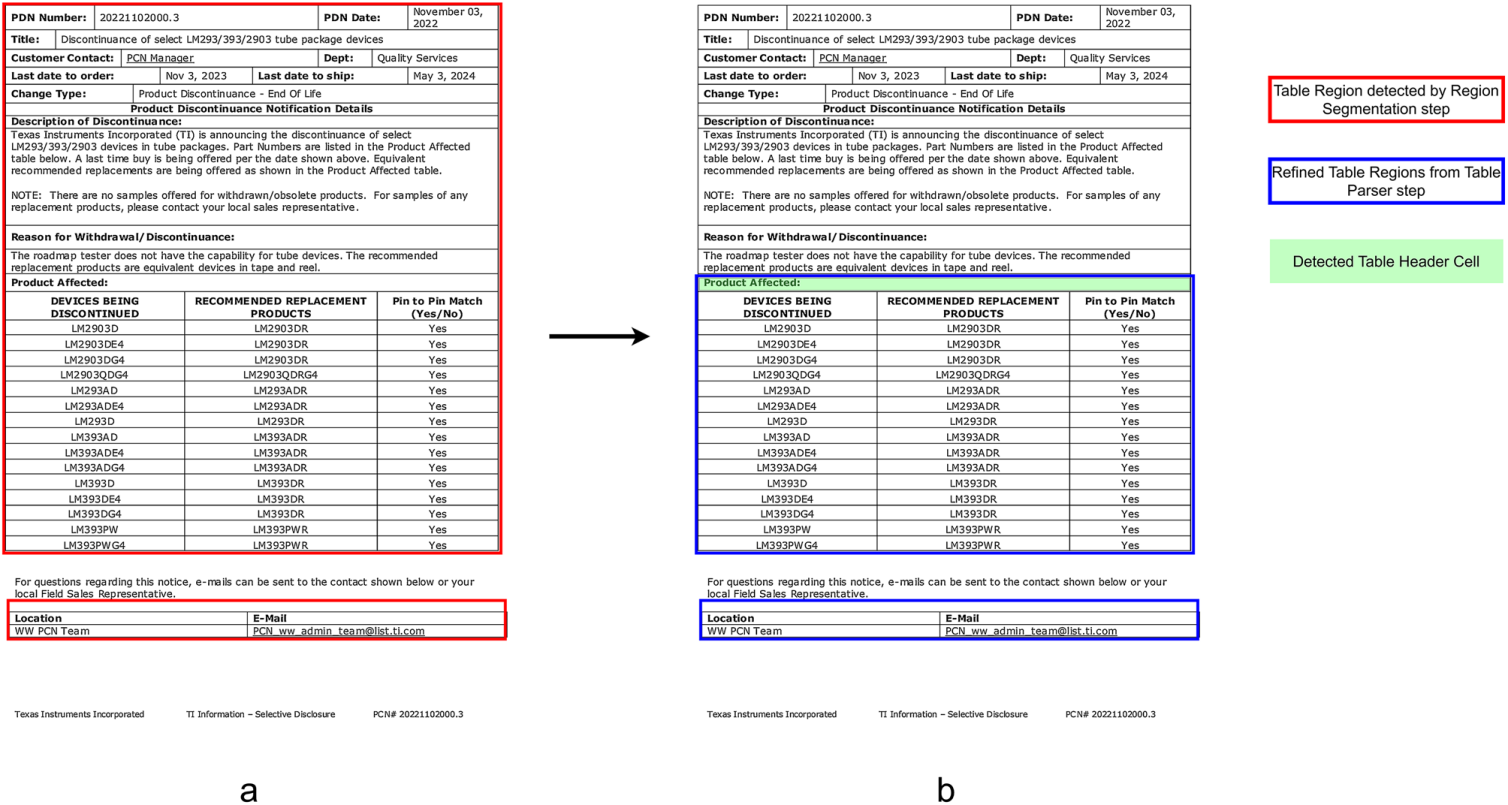
Refined table regions from table parser (**b**) from the original table regions detected by the region segmentation step (**a**).

This problem is addressed by the table parsing step. In this step OpenCV’s line segment detector is used to operate directly only the gray-scale intensities which helps recover sub-pixel-level accurate segments that survive rotation or mile skew. This is done for both the horizontal and vertical line segments as shown in Fig. 10.

Once all these line segments are found, all the horizontal line segments that share same y-axis co-ordinates are merged into a single line segment. Also, all the vertical line segments that share the same x-axis co-ordinates are merged into single line segments. This method overcomes any skewness, rotations or breakage of boundaries caused by poor image quality or noise. After this process if there is any line segment that was not part of any merging process is discarded, as they might be false positives like an underline below certain text or etc.

Once these refinements are done and we have solid reliable table boundaries, we look for a grid structure again that represents a valid table structure. This again helps to refine the true table regions out of the original table region detected by the region segmentation step which sometime might have extra content that would have slipped in as a part of table region. This is done by identifying the group of cells that are vertically stacked and have almost similar width to form a valid column and then these group of valid columns sharing the same y-axis co-ordinates for the top edge to form a valid table. Thus, as a result we finally have a refined table region which forms as a reliable foundation for the next step.

#### Data curation

The main function of this step is to create a digital copy of the table and prepare it for OCR. One of the main components of this process is to identify the complex features of the tables like the presence of nested column structures and identifying the spanning cells. The Data Curation modules categories tables into the following types:

- Key-Value Table: The tables that have one of the columns as the labels or keys and the other as the values associated with those keys.
- Nested-Column Table: The tables that have nested column headers, for instance one parent column header encompassing more than one child column headers.
- Normal Table: The typical table type with columns and rows.

These are necessary to identify as they define how the OCR action will take place for these tables. The Key-Value Tables are identified if they follow the following criteria:

- If there are only two columns in the table.
- If the width difference between the two columns is significant.
- If the total number of rows is less than equal to 5.

**Fig. 11:**
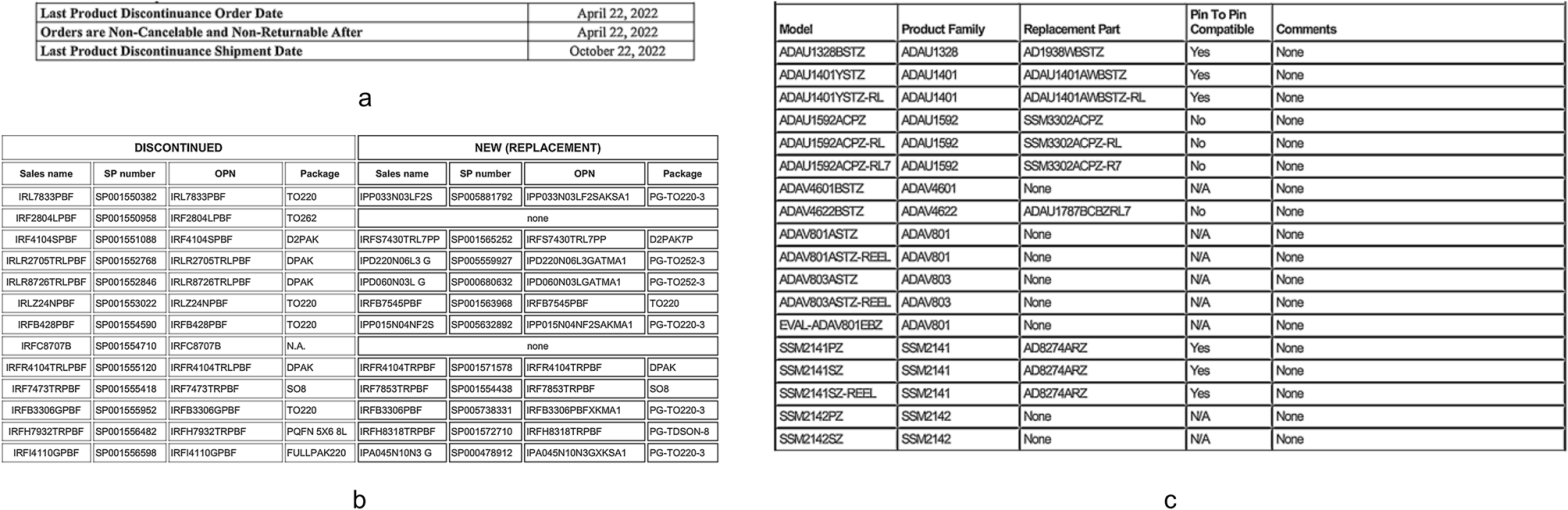
Examples of different types of tables (**a**) key-value table, (**b**) nested column table and (c) normal table.

As describe in Fig. 11, the tables that satisfy these criteria and marked as Key-Value Tables. But the most challenging part of these modules is to identify the nested column structures and the spanning or merged cells. To address these challenges few clever strategies have been undertaken.

**Fig. 12:**
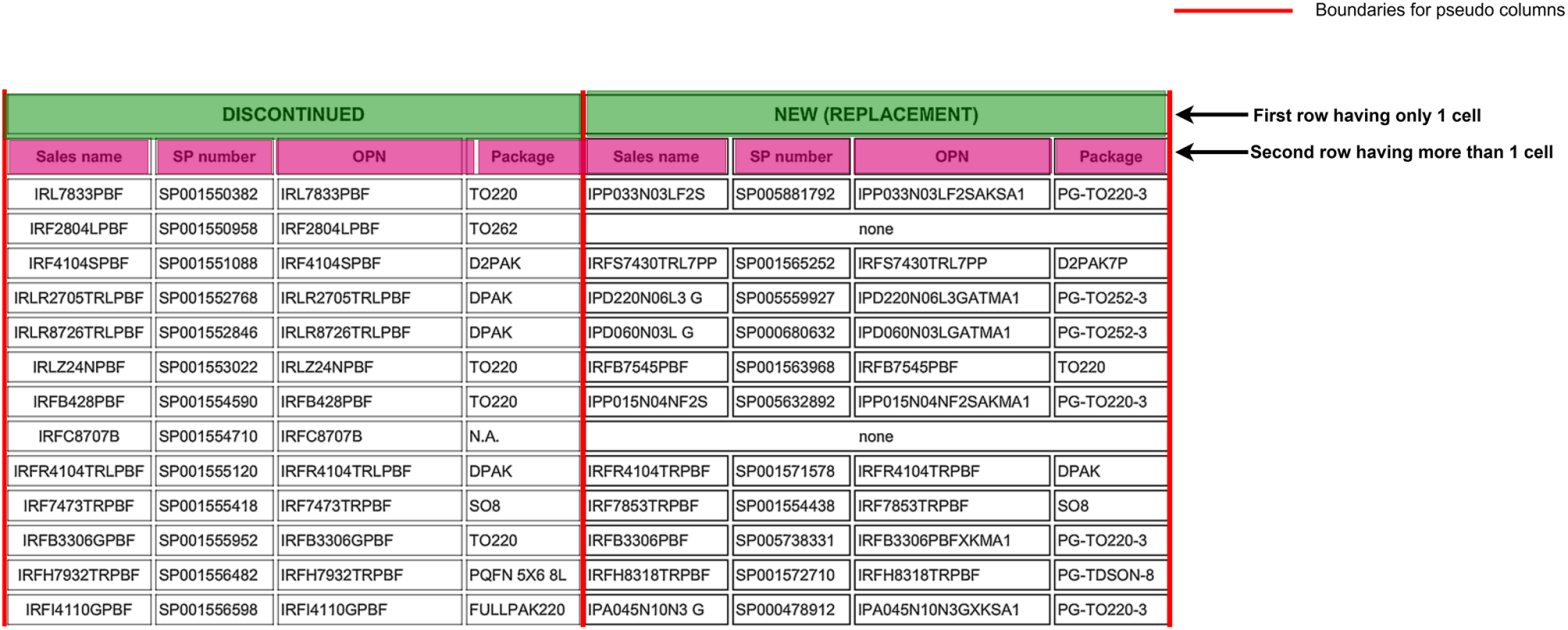
Detection of nested column structure.

Since we have already got a highly reliable cell, column and row data from the table parser step and also since the table segment image is literally cropped to the table-region, we utilize them to aid these challenges. The very first step is to identify if there is any header cell, this is fairly easy as the header cells will usually span the entire width of the table which is our case is entire image. Thus, once the header cell is identified we discard it out from any of the further considerations until OCR step. Then we look for the largest line vertical line segments that share the same y-axis co-ordinates in the given table. For now, we call them pseudo columns. If the table has a nested column structure, then we will find that the first row has less number of cells than the second row. This clearly marks the presence of a hierarchical structure within the pseudo column. We check this top-down until we the number cells don’t change from one row to another. As an additional check we also check that if the edges of the parent column cells are aligning with that of the child column cell ensuring the spanning of the parent column cell is uniform. Once these properties are identified, we mark that table as nested column structure, and the nesting data is stored accordingly in appropriate data structure as depict in Fig. 12.

To Detect Spanning Cells, we take all the horizontal line segments (which here will always be top and bottom edge of the all the cells in same row) and the all the vertical line segments (which here will always be left and right edge of all the cells in same column) and extend them to the entire width and height respectively. For a normal table all these extended line segments should pass through the edges of all the cell literally making no difference. But if the cell is a merged cell or in other words is a spanning cell then then some of these extended lines will pass through the inside of the cell rather than passing through the edges. For a cell which is merged across multiple rows, will have multiple horizontal lines passing through it and for the cells which are merged across multiple columns will have multiple vertical lines passing through it and for the cell which is merged across both rows and columns will obviously have both horizontal lines and vertical lines passing through it.

As directly calculating the height and width of a cell and comparing it to the other adjacent cells for determining if the cell is spanning or not is rather complex and unreliable strategy, our proposed method really comes in handy to provide more reliable, explainable and deterministic heuristics to define spanning cells. Moreover, we can determine the number of rows or columns a particular cell is spanning by just simply calculating span = (the number of horizontal or vertical lines passing through it + 1).

In the final data structure, we create a digital copy of the table by storing the bounding co-ordinates of each cell arranged with respect to the columns and rows that they belong in. For the merged cell we simply repeat the bounding box co-ordinates in the data structure for every row or column that the cell is spanning across. The data structure also stores other meta data about the table like the type of table as discussed in the beginning and at cell level stores data if the cell is merged or not, if merged then what kind of merged: row/column/both? This helps determine how the OCR process will behave for this table.

**Fig. 13:**
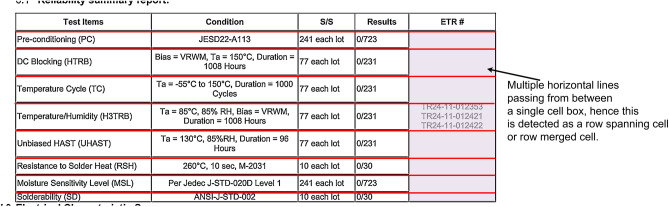
Detection of merged cell.

Once we have identified these complex features out of the tables, we move towards the OCR step to extract data out of the table. In the OCR step we have employed two different strategies, one is Single Cell OCR and other is Multi Cell OCR as shown in Fig. 13.

##### Single cell OCR

**Fig. 14:**
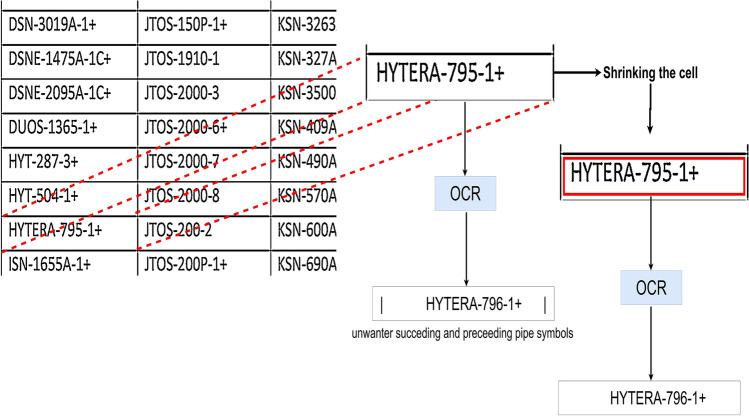
Depiction of how shrinking the cell in the single cell OCR strategy improves the OCR quality.

As the name suggests, the single cell OCR performs OCR on the cell level i.e. we extract text from each cell one by one and arrange it in its logical order. This strategy ensures high quality data extraction as there is no extra noise as image that goes under the OCR process is cropped to the cell boundaries. We also shrink the cell by 3 pixels on each side so that the actual boundaries of the cell don’t go under the OCR process which may yield unwanted text like “|” or “!” before or after the actual text as shown in Fig. 14.

##### Multi cell OCR

**Fig. 15:**
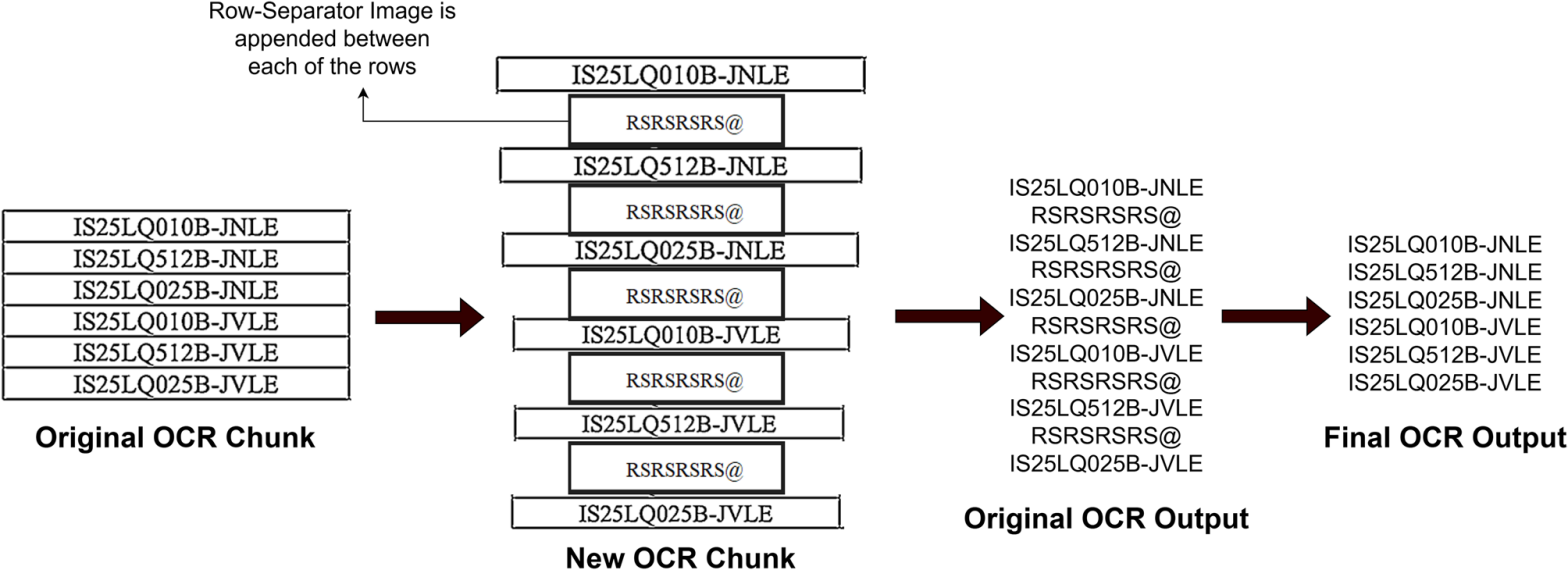
Depiction of multi cell OCR strategy and appending of the row-separator image, and splitting the original OCR output based on the row-separator text to get the final OCR output.

As depict in Fig. 15, Multicell OCR chunk the cells in a column in the group of 10, 15 or 20 (this value is decided by the user at the start of the program) and perform OCR on these chunked cells at once.

Now if we simply extract these text and separate different rows just splitting based on the new line, a single cell having text in multiple lines will get split into multiple unwanted rows. To overcome this problem, we append our own custom image row-separator image between each cell of the chunking group. This image contains the text “RSRSRSRS@”. This text was decided after multiple trials and errors as the OCR almost always extracted this text with 100% precision. Thus, we split the chunked text based on our row-separator text into separate rows. This ensures that no unwanted rows are created because of newline characters in a single cell. As a final sanity check we also check if the number of rows extracted from OCR is equal to the value of total number of cells stored in the data structure from the previous steps.

**Fig. 16:**
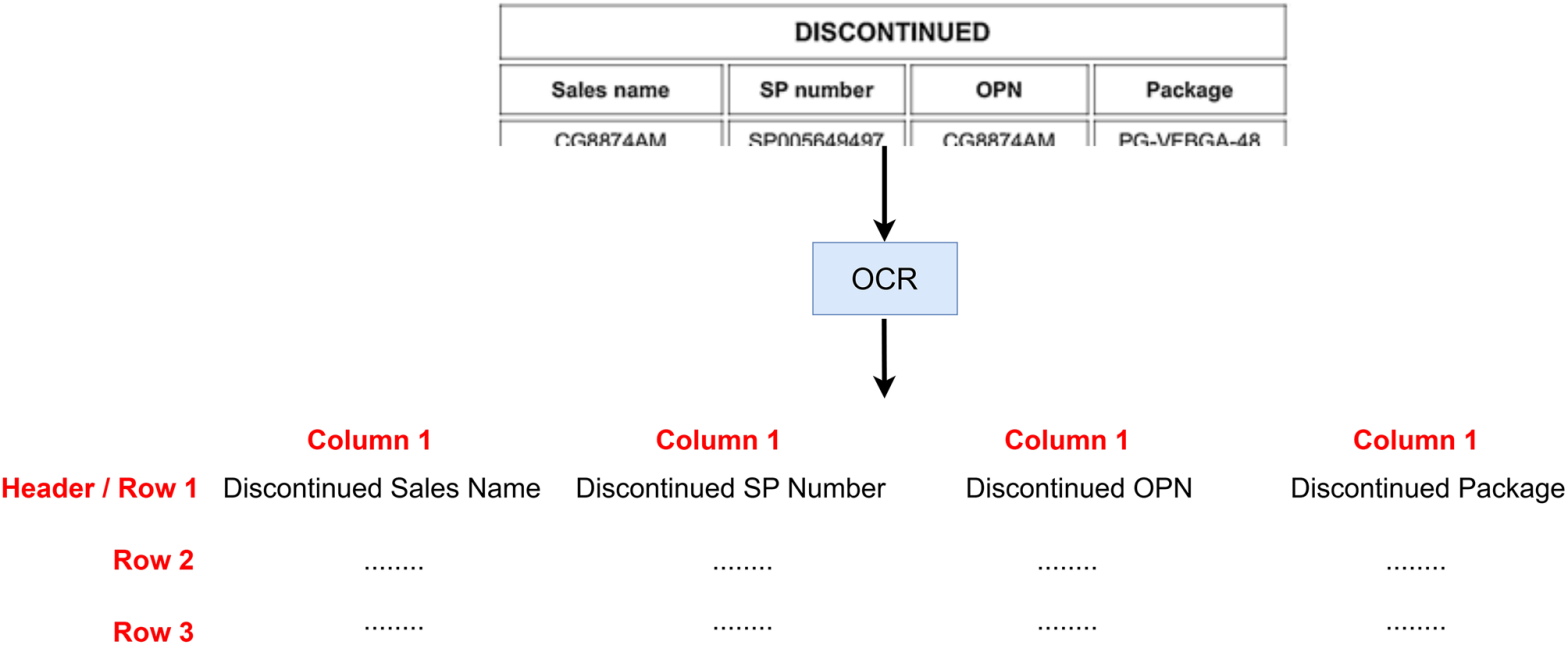
OCR Extraction for nested column tables.

As shown in Fig. 16, for nested column table the values of parent–child columns are merged from parent to child and thus it becomes a single header. For example, if the parent column value is “Discontinued” and the child columns are “Parts”, “Package” and “OPN”, then while doing the OCR we will merge them so that they become “Discontinued Parts”, “Discontinued Package” and “Discontinued OPN”. This helps in simplifying further process and maintaining a uniform structure of the final JSON that is created per page without losing the hierarchical significance of nested columns. If the chunking encounters a cell that is marked as merged cell, then this cell is left alone, and chunking happens around this cell. The merged cell is extracted via the single cell OCR strategy as passing this through the simple multi cell strategy can cause misalignment of data. Although it must be noted that more the number of cells chunked together, lesser the extraction accuracy.

Whether to use the single cell OCR strategy or the multi cell one can be decided by us at the beginning of the program. We also have an “auto” configuration where the program automatically switches between single cell or multicell based on the total number of cells in the table. If the total number of cells is less than 120, then single cell else switch to multi cell.

We use Tesseract OCR as standard as it is lightweight, open source and runs offline. But we can easily swap any other OCR model if a better model is available at our disposal. The pipeline structure was thus kept modular so that the given OCR engine does not become the limiting factor to the OCR quality as we can always switch to better model anytime.

After OCR is done, we merge the similar tables on the given page. If the two tables in the give page have same number of columns and width of each column is also almost same for these two tables, or in case of presence of table header, if both the table headers are same, we combine these tables into one table. Finally, we create a structured JSON containing all the data of tables and other segments (Pre-Text, Inter-Text and Post-Text) accordingly.

### Post-processing

In this step we give a final shape to the JSON data collected from all the pages of the document before presenting it as final output. At this point we can either choose to just convert all the extracted tables into CSV files and end the process or we can continue with document level merging process.

#### Merging across pages and creating a single final JSON

The JSON data created per page is examined and then merged into one single JSON representing the complete document. We merge the similar tables based on the column header values, number of columns, columns widths and table headers just like we did for per page merging.

#### The LLM hook

Optionally we can plug in LLM APIs like the ones available for the chat completion models provided by OpenAI to fetch only the table headers or columns headers that are required by us while ignoring the others. This can be done by describing the model via a prompt about the type of column headers or table headers that we want to extract and the LLM can give an output that contains the names or indices of that column headers according to our JSON structure by which we can ultimately fetch the required columns or tables.

We could also provide all the text regions like pre-text, post-text or inter-text to the LLM and fetch any specific meta data that we want to fetch alongside the tables. This lets us process our documents holistically rather than sticking to just table detection or extraction.

## Results and discussion

We have systematically evaluated the performance of the SPARTAN methodology critically, with emphasis on its practical efficiency and robustness in handling different table structures in PDF documents. For a realistic testing setup and a valid test, SPARTAN was tested on a variety of datasets, mainly Product Change Notifications (PCNs) and Product Discontinuation Notifications from well-known electronic component manufacturers (PCN-480 dataset). The tests were also done on scientific papers and typical document types, further expanding the scope of our performance tests.

### Evaluation criteria

To quantify both extraction quality and deployability, we targeted six measures: precision, recall and their harmonic mean (F1) quantify how reliably SPARTAN distinguishes true table areas; OCR character‑accuracy widens the focus from geometry to content, indicating whether the detected cells yield machine‑readable text; and the set of processing‑time and peak RAM plots the real-world cost of running the system at scale. Collectively, these measures paint a concise but thorough picture of performance in line with the statistics reported for conventional baselines such as Tabula, TabbyPDF, Deepdoctection and EMbTTBF.

Graph-similarity measures like TEDS and GriTS were not considered because they need time-consuming cell-adjacency ground truth, penalize accurate benign cell fusions that actually improve OCR, and produce scores (e.g., 0.82 → 0.87) that are difficult for engineers to translate to risk or resource planning. Therefore, by focusing on the measures demonstrated in the following table, we have optimal interpretability, maintain minimal annotation burden, and provide direct comparability to previous work—while still varying across the full range from detection fidelity to computational efficiency as describe in Table 4.

**Table 4:** Metric suite used to evaluate SPARTAN.

Metric	Aspect measured	Practical rationale
Precision (P)	False‑positive control	Avoids over‑segmentation in multi‑box documents
Recall (R)	False‑negative control	Ensures completeness for analytics pipelines
F1‑score	Balance of P & R	Single figure for cross‑paper comparison
OCR accuracy	Text fidelity	Links geometric detection to usable data
Processing time	Throughput	Determines batch‑processing feasibility
Peak RAM	Footprint	Governs container sizing & cloud costs

All the metrics have been described as follows:

- Precision (P): Accuracy in identifying actual table structures without false positives.

$$P = \frac{True Positive}{{True Positive + False Positive}}$$

- Recall (R): Ability to detect all actual table structures without omission.

$$R = \frac{True Positive}{{True Positive + False Negative}}$$

- F1-Score (F1): Balanced harmonic mean of precision and recall.

$$F1 = 2 \times \frac{{\left( {P \times R} \right)}}{{\left( {P + R} \right)}}$$

- OCR accuracy

$$OCR Acc = 1 - \frac{Character Errors}{{Total Characters}}$$

- Processing time: Average processing time per page, to measure computational efficiency.
- Peak RAM: Maximum resident set measured by psutil.Process.memory_info().rss [“-“ after Pro is just to indicate continuation and is not intended].

### Implementation details

While the implementation for the testing purpose, SPARTAN had the following OCR configurations:

- OCR Model → Azure Computer Vision OCR.
- OCR Scope → auto: Single for total number of cells < 120, 15 cell chunked if the total number of cells > 120.

This is important to mention because the OCR configuration can greatly affect the overall processing time of the whole document in SPARTAN. We found that using the single cell OCR strategy for all documents or to be precise for any number of total cells results in increasing processing time by 65% with additional factors like the internet speed as the Azure Computer Vision OCR is a cloud-based service. Thus, the auto configuration is a significant time leap over the purely single cell OCR strategy. This does not mean to prove that single cell strategy is completely useless as it has its own strength when the OCR accuracy is at the priority but one should also be mindful of its limitations in terms of processing time and thereby use this strategy smartly. The auto configuration is an attempt to do just that.

We processed multiple PDF through SPARTAN, having different number of pages and calculated the processing time taken to process each of those PDFs as shown in Table 5.

**Table 5:** Processing time calculations of PDF with different total page numbers.

No. pages in PDF	Total processing time (seconds)	Average processing time = (Total processing time / total number of pages in PDF)
1	4.39	4.39
2	8.72	4.3
4	17.16	4.29
5	21.95	4.39
10	43.07	4.307
15	63.74	4.24
20	87.1	4.35
30	124.19	4.13
40	164.34	4.10

The implementation revealed that the processing time grew linearly with the increase in the total number of page numbers with an average processing time of approximately 4.2 s per page.

**Fig. 17:**
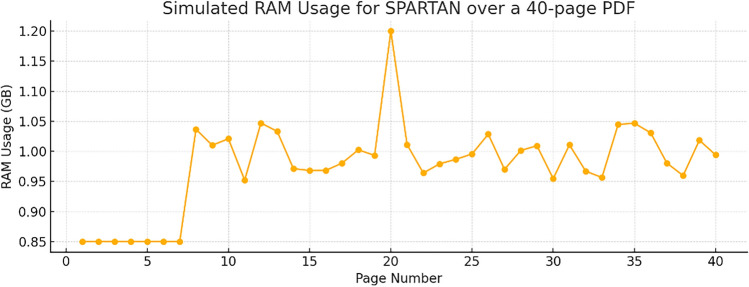
RAM usage for the runtime of the 40-page PDF showing the striking of Peak RAM usage by SPARTAN during the entirety of testing.

For the entirety of the testing (Fig. 17), SPARTAN reached a peak RAM usage of 1.2 GB which occurred around the 20th page of the 40-page PDF, indicating a dense table (Table having lot of cells in a single page) around those pages. The following snapshot shows the RAM usage for the entire runtime of processing the 40-page PDF.

All the testing was done on the system that was having following specifications:

- Processor: 11th Gen Intel(R) Core(TM) i5-1145G7 @ 2.60 GHz 2.61 GHz.
- RAM: 16.0 GB
- Operating System: Windows 10 Enterprise.

### Results comparison

Our SPARTAN module was tested against several other leading table extractors, including:

- Tabula
- Deepdoctection
- TabbyPDF^2^
- EMbTTBF

Please note that all these tools were tested on the same datasets on which the SPARTAN was tested. Also, it must be noted that the “Column Detection” Step had been turned off as most of the PDFs were single columned while testing. This was also important to conduct uniform and comparable testing among the given tools as not all of them have capabilities to deal with PDFs having columned layout. After performing all the tests, the following results were observed:

Table 6 demonstrates that SPARTAN has the highest precision (0.94), recall (0.91) and thus the highest F1‑score (0.93) of all systems, and the highest OCR accuracy (96.7%) as well. That is, SPARTAN not only detects more actual tables than Deepdoctection but also adds fewer false positives than Tabula, and the extracted text is the cleanest of the lot. This performance vindicates the use of the dual‑detector plus OCR‑optimized architecture in terms of measurable downstream benefits.

**Table 6:** Comparative analysis.

Model	P	R	F1	OCR Accuracy (%)
SPARTAN	**0.94**	**0.91**	**0.93**	**96.7**
Tabula	0.88	0.66	0.75	91.5
Deepdoctection	0.92	0.83	0.87	95.6
TabbyPDF^2^	0.89	0.79	0.84	93.4
EMbTTBF	0.91	0.86	0.88	94.7

Significant values are in bold.

#### Computational efficiency

**Fig. 18:**
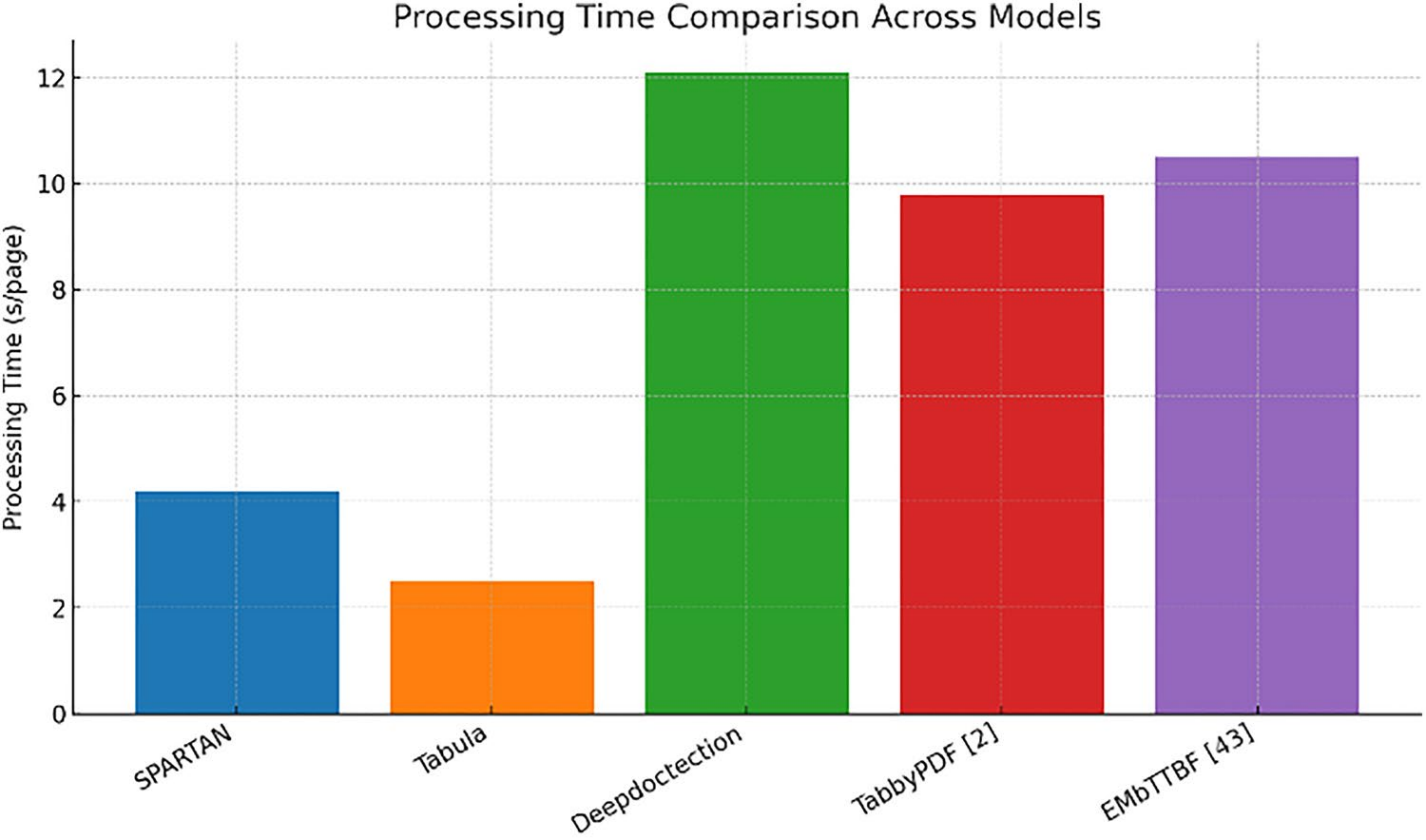
Graph comparing the processing time of each model.

As depict in Fig. 18 the processing time bar chart indicates that SPARTAN processes a page in ≈ 4 s, the second-fastest time after Tabula’s lightweight extractor but three times faster than Deepdoctection. With the enhanced quality metrics highlighted above, SPARTAN is at the optimal accuracy-to-latency spot on the Pareto front—a critical issue for batch workloads.

#### Memory footprint

**Fig. 19:**
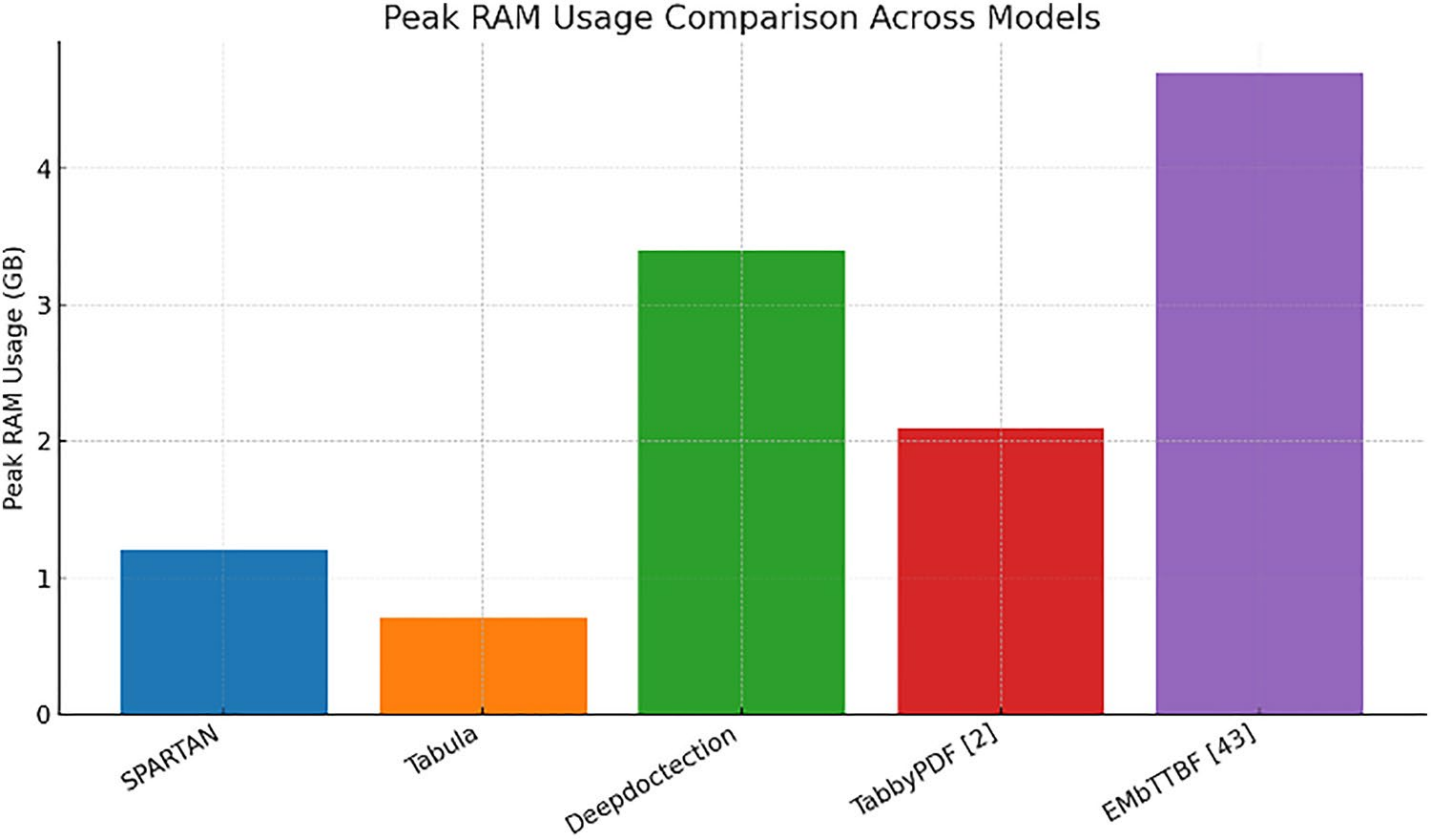
Graph comparing the peak-RAM Usage of each model.

The Fig. 19 shows peak‑RAM plot tracks a similarly consistent trend to the time plot: Tabula is once more the most conservative (0.7 GB), but SPARTAN is still humble at ~ 1.2 GB, while learned models shoot far above the 3 GB threshold. The consistent ordering on both plots—Tabula < SPARTAN ≪ {TabbyPDF, Deepdoctection, EMbTTBF}—indicates that SPARTAN’s heuristics scale in a consistent manner in both time and space, further supporting its appropriateness for resource‑limited deployment use cases.

We explicitly analyze SPARTAN’s behavior on out-of-distribution layouts and identify its primary failure cases. Performance degradation is observed mainly on low-quality scanned documents, severe noise or skew, and tables with highly irregular or decorative layouts that violate geometric alignment assumptions. In such cases, SPARTAN tends to behave conservatively, prioritizing precision over recall and avoiding false-positive table detections. This analysis clarifies SPARTAN’s cross-domain behavior and delineates the conditions under which its heuristic assumptions are most effective or may require additional preprocessing.

### Discussion

The SPARTAN evaluation had a compelling lead in structurally complex table processing. Unlike rule-based systems such as Tabula, which mis-segmented non-ruling-line layouts or simply dropped nested headers, SPARTAN’s two-detector heuristics consistently detected row and column boundaries even in the absence of ruling lines. Moreover, its pseudo-cell boundary overlays smoothly resolved merged and spanned cells, thereby allowing the parser to preserve the logical grid, while the competing tools produced fragmented cells or duplicate text. As a result, recall substantially improved and structural errors considerably fell for intricate layouts, thereby validating the strength of well-considered, geometry-sensitive heuristics.

Accuracy enhancements were facilitated by the OCR layer. Replacing Tesseract with Azure Computer Vision cut down on the common mis-reads (e.g., "I/1/!" errors), raising the character-accuracy rate to 98.7%. A bespoke multi-cell window—batch-reading 15 consecutive cells—insulated the recognizer from edge noise and reduced API calls, maintaining accuracy while increasing throughput. Combined with lightweight image processing, this architecture delivered page-level runtimes three-to-four times quicker than deep-learning pipelines such as Deepdoctection and EMbTTBF, an advantage that is critical to real-time or resource-constrained deployments.

Of comparable significance is SPARTAN’s modularity and open-source design. Users can switch between OCR engines, alter spacing and threshold heuristics, or insert new preprocessing operations without having to retrain a monolithic, fixed model, hence guaranteeing versatility across various document types and budgets of hardware. Collectively, the experiments demonstrate that a contemporary heuristic pipeline-complemented with high-quality OCR-can rival or beat the performance of deep-learning models and remains transparent, low-cost, and universally accessible, hence confirming SPARTAN’s contribution to table-extraction technology in practical application.

SPARTAN is fundamentally a table-detection and structure-extraction system; OCR is an optional downstream component. The contributions of SPARTAN, region segmentation, dual-path heuristics, nested-column detection, and cell-structure recovery, operate entirely before OCR is invoked. To ensure comparability with modern OCR-backed pipelines used in existing literature, Azure OCR was chosen for benchmarking. This maintains fairness when comparing SPARTAN against state-of-the-art systems that also rely on strong OCR engines.

Deep-learning baselines were not evaluated with GPU or optimized inference backends, and that reported runtimes therefore reflect out-of-the-box CPU inference performance.

## Future scope and conclusion

This research introduced SPARTAN, a new heuristic-based approach utilizing the latest OpenCV image analysis heuristics to identify and extract tables from different types of documents. Despite SPARTAN performing better within its current setup, there are potential directions towards further enhancement, wider application and integrative approaches, hence opening various future research and development avenues. The limitation of SPARTAN is.

### Future scope

A potential first extension step is to add to SPARTAN’s purely heuristic core certain deep-learning pieces that are specialized in the pathologies of scanned or camera-scanned pages. Lightweight CNN de-skewers, denoisers and super-resolution modules would be called only when the input is bitmap-based, maintaining the overall CPU frugality of the pipeline and just improving recall on low-quality scans. Together with careful GPU or multi-core parallelization, the same architectural hooks open the door to real-time streaming: SPARTAN could consume sustained PDF batches from finance back-offices, logistics hubs or emergency feeds and produce structured data within milliseconds, within the latency budgets of the decision-support dashboards.

A second strategic area is service-based delivery. Encasing SPARTAN in a stateless REST API and running it on elastic infrastructure (Azure Functions, AWS Lambda, Google Cloud Run) would bring it within reach of organizations without on-prem compute. Auto scaled GPU pools for the deep-learning optional steps are also supported by cloud orchestration and expose a plug-in interface for extended semantic post-processing. For instance, large language models like GPT-4 Turbo or LLaMA 3 can label columns, generate natural-language summaries of numeric tables, and expose implicit metadata as JSON—rendering SPARTAN a full document-understanding micro-service. Finally, portability and community-sourced power features will continue to be relevant in the long run. An edge-optimized build developed using ONNX-Runtime or TensorRT and optimized specifically for ARM chipsets would allow field techs, warehouse scanners, or disaster-relief personnel to natively read forms on their mobile devices without any network access. Having the codebase maintained on GitHub under a permissive license enables researchers and practitioners to contribute heuristic tweaks, language packs, or even new detection branches through pull requests, essentially ensuring that SPARTAN adapts with the changing document format and industry needs.

### Conclusion

Effective extraction of structured data from documents has increasingly been a vital issue in various industries and research domains. Even with tremendous advancements, current methods still suffer from considerable limitations in terms of computational demand, accessibility, and their ability to accommodate varying document structures. Overcoming these major issues, SPARTAN is a significant effort in the area of automatic table detection and extraction with a strong, adaptable, and computationally efficient solution achieved by carefully designed heuristics, sophisticated image processing algorithms, and improved optical character recognition integration.

The comprehensive evaluations carried out in this thesis have demonstrated SPARTAN’s improved performance in accurately detecting and extracting tables from complex layouts, including borderless, nested, and irregular ones. SPARTAN outperformed current methods, such as Tabula, Deepdoctection, TabbyPDF, and EMbTTBF, on key metrics like accuracy, recall, OCR accuracy, and processing time. This robust performance supports SPARTAN’s applicability, effectiveness, and reliability in real-world document processing scenarios.

Besides, the distinctive modular design of SPARTAN, the ease of its customization and the open-source nature greatly improve its usability and flexibility. This aspect makes it a great asset not only for technical specialists but also for the broader community with limited computational capabilities or technical knowledge. Its ability to be paired with powerful OCR services, like Azure Computer Vision, has contributed greatly to accuracy and reliability, raising the bar in heuristic-based document processing tools.

In conclusion, SPARTAN adequately addresses the significant shortcomings that have been cited in existing literature, thus rendering the heuristic-based methods applicable and relevant in modern document processing. With additional research and cooperation within the community, SPARTAN can continue to advance, mature, and expand its capabilities, thus remaining applicable and relevant across different industries and research fields.

## Data Availability

The datasets analysed during the current study are available in the Product Change Notifications (PCNs) repository, [https://www.latticesemi.com/en/Support/PCN].
